# Supplementary material for: Multi‐dataset identification of innovative feature genes and molecular mechanisms in keratoconus
Source: J Cell Mol Med. 2024 Sep 19;28(18):e70079. doi: 10.1111/jcmm.70079 (PMC11412914; doi:10.1111/jcmm.70079)
Supplement: Supplementary file 2 — Material S2. [file JCMM-28-e70079-s002.zip › 7.GSEA analysis/1.ARL11/KEGG_PRIMARY_IMMUNODEFICIENCY.html]

Details for gene set KEGG\_PRIMARY\_IMMUNODEFICIENCY[GSEA]

|  || Dataset | ARL11.group.cls#h\_versus\_l |
| Phenotype | group.cls#h\_versus\_l |
| Upregulated in class | h |
| GeneSet | KEGG\_PRIMARY\_IMMUNODEFICIENCY |
| Enrichment Score (ES) | 0.39954913 |
| Normalized Enrichment Score (NES) | 1.1052454 |
| Nominal p-value | 0.3212237 |
| FDR q-value | 1.0 |
| FWER p-Value | 0.996 |
Table: GSEA Results Summary

  

Fig 1: Enrichment plot: KEGG\_PRIMARY\_IMMUNODEFICIENCY      
 Profile of the Running ES Score & Positions of GeneSet Members on the Rank Ordered List

  

| SYMBOL | TITLE | RANK IN GENE LIST | RANK METRIC SCORE | RUNNING ES | CORE ENRICHMENT || 1 | IL7R | na | 136 | 0.619 | 0.1205 | Yes |
| 2 | CD8B | na | 580 | 0.460 | 0.2002 | Yes |
| 3 | PTPRC | na | 886 | 0.402 | 0.2724 | Yes |
| 4 | CIITA | na | 1433 | 0.343 | 0.3257 | Yes |
| 5 | JAK3 | na | 1845 | 0.308 | 0.3757 | Yes |
| 6 | CD19 | na | 3009 | 0.240 | 0.3904 | Yes |
| 7 | CD3E | na | 4816 | 0.171 | 0.3729 | Yes |
| 8 | ZAP70 | na | 5041 | 0.165 | 0.3995 | Yes |
| 9 | ADA | na | 6434 | 0.126 | 0.3847 | No |
| 10 | CD3D | na | 6811 | 0.116 | 0.3972 | No |
| 11 | RAG1 | na | 8315 | 0.086 | 0.3713 | No |
| 12 | CD8A | na | 8817 | 0.078 | 0.3725 | No |
| 13 | UNG | na | 9899 | 0.061 | 0.3536 | No |
| 14 | DCLRE1C | na | 9958 | 0.061 | 0.3642 | No |
| 15 | ICOS | na | 10447 | 0.054 | 0.3611 | No |
| 16 | CD4 | na | 11083 | 0.047 | 0.3521 | No |
| 17 | BLNK | na | 11271 | 0.044 | 0.3557 | No |
| 18 | RFXAP | na | 13286 | 0.025 | 0.3026 | No |
| 19 | LCK | na | 13290 | 0.025 | 0.3074 | No |
| 20 | RFXANK | na | 14977 | 0.011 | 0.2611 | No |
| 21 | CD79A | na | 15111 | 0.010 | 0.2593 | No |
| 22 | TAP2 | na | 16908 | -0.003 | 0.2081 | No |
| 23 | IL2RG | na | 20265 | -0.026 | 0.1165 | No |
| 24 | TAP1 | na | 20591 | -0.028 | 0.1128 | No |
| 25 | TNFRSF13C | na | 20657 | -0.029 | 0.1167 | No |
| 26 | RFX5 | na | 22026 | -0.040 | 0.0853 | No |
| 27 | AIRE | na | 22124 | -0.041 | 0.0908 | No |
| 28 | IKBKG | na | 22799 | -0.048 | 0.0810 | No |
| 29 | AICDA | na | 24863 | -0.074 | 0.0365 | No |
| 30 | CD40 | na | 26388 | -0.096 | 0.0119 | No |
| 31 | IGLL1 | na | 27304 | -0.115 | 0.0086 | No |
| 32 | RAG2 | na | 27380 | -0.116 | 0.0299 | No |
| 33 | BTK | na | 31389 | -0.235 | -0.0384 | No |
| 34 | CD40LG | na | 31605 | -0.244 | 0.0045 | No |
| 35 | TNFRSF13B | na | 34040 | -0.425 | 0.0198 | No |
Table: GSEA details [plain text format]

  

Fig 2: KEGG\_PRIMARY\_IMMUNODEFICIENCY      
 Blue-Pink O' Gram in the Space of the Analyzed GeneSet

  

Fig 3: KEGG\_PRIMARY\_IMMUNODEFICIENCY: Random ES distribution      
 Gene set null distribution of ES for **KEGG\_PRIMARY\_IMMUNODEFICIENCY**
